# Molecular mechanisms of Zika virus teratogenesis from animal studies: a systematic review protocol

**DOI:** 10.1186/s13643-021-01713-6

**Published:** 2021-05-29

**Authors:** Gabriela Elis Wachholz, Julia do Amaral Gomes, Juliano André Boquett, Fernanda Sales Luiz Vianna, Lavínia Schuler-Faccini, Lucas Rosa Fraga

**Affiliations:** 1 Graduate Program in Genetics and Molecular Biology, Department of Genetics, Biosciences Institute, Universidade Federal do Rio Grande do Sul, Porto Alegre, 91501-970 Brazil; 2 Teratology Information Service, Medical Genetics Service, Hospital de Clínicas de Porto Alegre, Porto Alegre, 90035-903 Brazil; 3 Laboratory of Genomic Medicine, Experimental Research Center, Hospital de Clínicas de Porto Alegre, Porto Alegre, 90035-903 Brazil; 4 Graduate Program in Child and Adolescent Health, Faculty of Medicine, Universidade Federal do Rio Grande do Sul, Porto Alegre, Brazil; 5 Graduate Program in Medicine: Medical Sciences, Universidade Federal do Rio Grande do Sul, Porto Alegre, 90035-003 Brazil; 6 Department of Morphological Sciences, Institute Health Sciences, Universidade Federal do Rio Grande do Sul, Porto Alegre, RS 90050-170 Brazil

**Keywords:** Gene expression, Epigenetics, Experimental models, Microcephaly, CNS birth defects, Infection

## Abstract

**Background:**

Due to the diversity of studies in animal models reporting that molecular mechanisms are involved in the teratogenic effect of the Zika virus (ZIKV), the objective of the present study is to evaluate the methodological quality of these studies, as well as to demonstrate which genes and which molecular pathways are affected by ZIKV in different animal models.

**Methods:**

This search will be performed in four databases: PubMed/MEDLINE, EMBASE, Web of Science, and Scopus, as well as in the grey literature. The studies selection process will be reported through the *PRISMA Statement* diagram model. All studies describing the molecular mechanisms possibly involved in the development of malformations caused by embryonic/fetal ZIKV exposure in animal models with an appropriate control group and methodology will be included (including, for instance, randomized and non-randomized studies). All animals used as experimental models for ZIKV teratogenesis may be included as long as exposure to the virus occurred during the embryonic/fetal period. From the selected studies, data will be extracted using a previously prepared standard form. Bias risk evaluation will be conducted following the *SYRCLE*’s Risk of Bias tool. All data obtained will be tabulated and organized by outcomes (morphological and molecular).

**Discussion:**

With the proposed systematic review, we expect to present results about the methodological quality of the published studies with animal models that investigated the molecular mechanisms involved in the teratogenic effect of ZIKV, as well as to show the studies with greater reliability.

**Systematic review registration:**

PROSPERO CRD42019157316

**Supplementary Information:**

The online version contains supplementary material available at 10.1186/s13643-021-01713-6.

## Background

Zika virus (ZIKV) was first isolated from rhesus monkeys in the Zika Forest (Uganda) in 1947, and the first cases of human infection were reported in Nigeria in 1954 [6, 12]. In 2015, a large increase of newborns with microcephaly was reported in Brazil [13], and it is already consolidated by the literature that this increase was due to the exposure to ZIKV infection during pregnancy [13, 18, 19].

The most concerning effects of ZIKV infection are related to pregnancy, when its infection can lead to a set of abnormalities related to impaired Central Nervous System development of the embryo/fetus. The phenotype spectrum ranges from absent/mild/moderate microcephaly without distinctive dysmorphic features to a severe microcephaly, and also can include contractures, ranging from dimples to generalized arthrogryposis [5]. Such abnormalities comprise the Congenital ZIKV Syndrome (CZS) [4, 5].

Several experimental studies have presented results that not only confirm ZIKV as the causative agent of the observed congenital anomalies, but also suggest possible molecular mechanisms by which the virus leads to adverse outcomes. These animal studies include non-human primates, chicken embryos, hamsters, guinea pigs, and swine [3, 10, 14, 17, 23, 24], using different approaches to analyze the mechanisms of infection and teratogenesis of ZIKV. Such results are related to ZIKV changes in gene expression in genes involved in autophagy and apoptosis processes in mouse embryos’ brains [2] as well as cell cycle and immune response [11].

Little is known about how ZIKV can actually produce the visualized changes in affected babies. In vivo studies have pointed out the different pathways and genes or proteins that are involved in this process; however, there are few literature reviews on this field and no systematic reviews. Thus, to compile, the results from experimental models are important not only to understand the molecular mechanism of the syndrome, but also to propose hypotheses, prevention strategies, and possible treatment to the damage caused by the virus.

## Objective

The objective of this systematic review is to assess the methodological quality of the studies in animal models that investigated the molecular mechanisms of ZIKV teratogenesis. Through a systematic search in literature databases and proper data extraction and analyses, in addition to the evaluation of the methodological quality of the studies, we aim to present the genes and molecular pathways which were shown to be affected by ZIKV in different animals.

## Methods/design

### Study question

This systematic review protocol has the following research question: what are the molecular mechanisms involved in the teratogenesis of the Zika virus proposed in studies with animal models?

### Protocol and registration

This protocol was developed by the members of the Reproductive and Developmental Biology Laboratory at Universidade Federal do Rio Grande do Sul. These members are biologists and physicians specializing in clinical, molecular and experimental teratogenesis. This protocol was written in accordance with the Preferred Reporting Items for Systematic Reviews and Meta-Analysis Protocols (PRISMA-P) guideline [15, 20]. In addition, the protocol has been registered with the International Prospective Registry of Systematic Reviews (PROSPERO) under registration ID CRD42019157316 (https://www.crd.york.ac.uk/prospero/display_record.php?ID=CRD42019157316).

### Eligibility criteria

From the question proposed in this protocol, the study’s Population, Intervention, Comparison, and Outcome (PICO) was established, as well as its resulting concepts:

#### Population

The *population/species* studied will be animal models with Congenital Zika Syndrome, including all species already studied in all different types of biomedical and biological experimental studies regardless of gender or species.

#### Intervention

The *intervention/exposure* will be the exposure to ZIKV during the embryonic/fetal period of development in any method of exposure, in any viral amount/titration, and at any stage of the embryo/fetal development.

#### Comparison

The *control population* will be given by a group of animals in the same study which undergone the same conditions of the treated/exposed animals but without ZIKV or other virus exposures.

#### Outcome

Two *outcomes* will be assessed. A primary outcome is that the studies must be analyzing the occurrence of congenital anomalies in the ZIKV-treated animals, where at least one morphological parameter (anatomical, histological, etc.) proves such effects. A secondary outcome is that all studies must have analyzed the presence or absence of molecular changes caused by such exposure that may be correlated to the observed anomalies. These molecular parameters include gene expression, proteomics, methylation patterns, or any other approach that investigates the possible changes in the patterns, structure, or amount of cellular macromolecules (DNA, RNA and proteins).

### Search strategy

A literature search to find relevant studies will be performed in four databases: PubMed/MEDLINE, *EMBASE*, Web of Science and Scopus, as well as in the grey literature, through the following sources: bioRxiv, OpenGrey, and PQDT Open. The databases present publications from medical, biomedical, pharmacological and life sciences journal literature. Before starting this protocol, searches were performed in order to identify any existing systematic review of molecular mechanisms of ZIKV from animal studies.

A combination of controlled vocabulary terms [e.g., Medical Subject Headings (MeSH)] and free-text terms will be used in the searches. For instance, in PubMed/MEDLINE search for ZIKV, the following will be used: Zika Virus[mh] OR Zika Virus Infection[mh] OR NS1 protein, zika virus[nm] OR Zika[tiab] OR ZikV[tiab]. Drafts for the search strategies for all the four databases that will be used for the systematic review are provided in Supplementary Table 1. This list was developed by all the members of the research group after consulting a librarian with extensive experience in setting up search strategies for systematic reviews of the Universidade Federal do Rio Grande do Sul. No language restrictions or publication date will be applied.

### Study selection

Abstracts, conference papers, erratum, letters to the editor, and short survey will be considered for the systematic review. Review articles; editorials; case reports; studies with humans or human samples; studies exclusively ex vivo, in vitro, or in silico; studies in which methodological information is absent or unclear; and studies that have no relevance to the research question will be excluded. In addition, vaccine development studies will also be excluded. Studies in which the exposure to ZIKV has occurred in the postnatal period will not be considered.

**Fig. 1:**
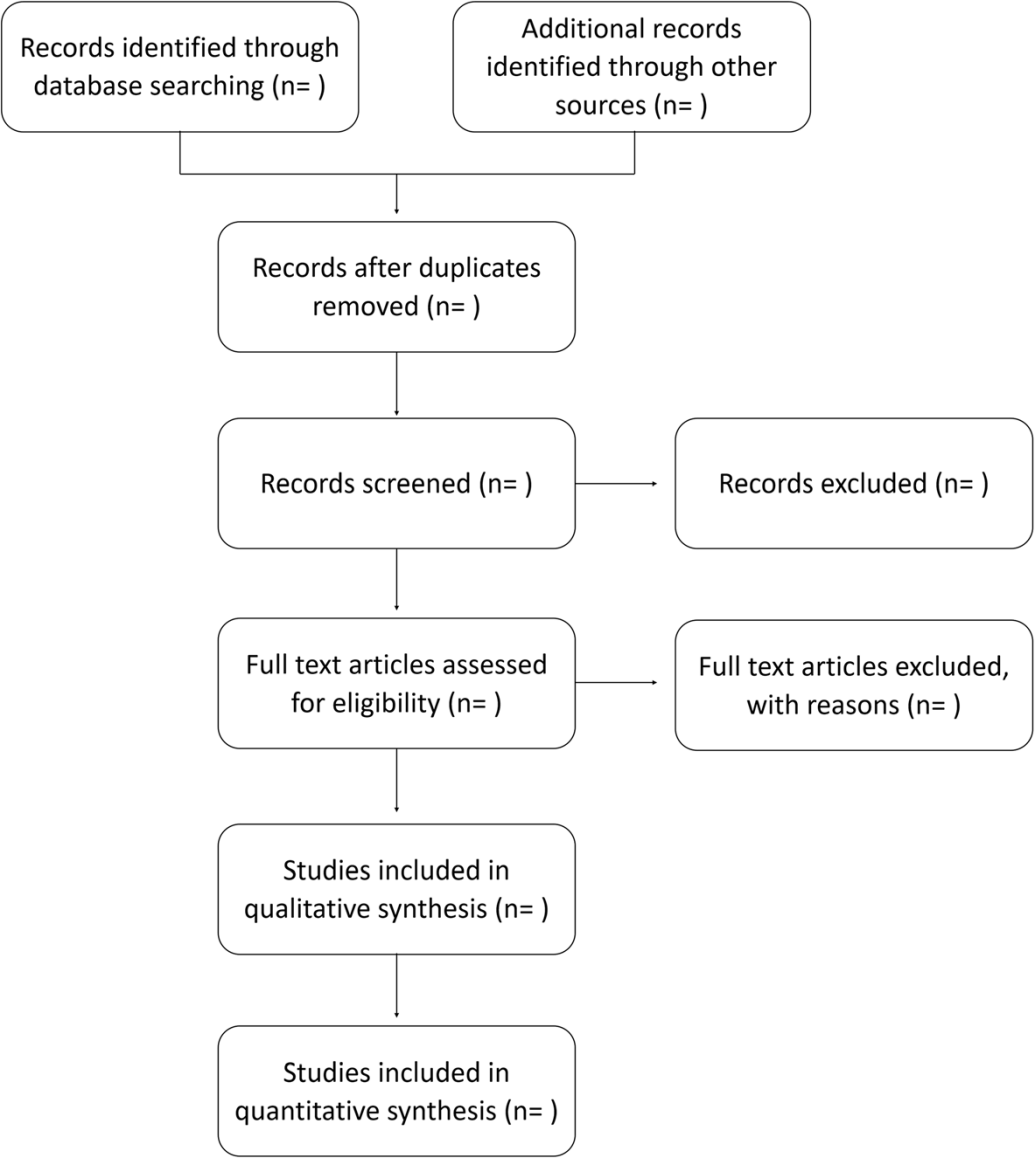
Flow chart showing the search and screening strategy to identify publications eligible for investigating the molecular mechanism of ZIKV teratogenesis from animal studies

The process from searching for studies to the final selection of studies that will compose the systematic review is shown in Fig. 1. The selection of the relevant studies will be performed in two phases. In the first phase, two independent reviewers will screen by reading the titles and abstracts of the articles. In the second phase, full texts of the articles selected in the first phase will undergo an evaluation. The study selection process will be reported through the *PRISMA Statement* diagram model (Fig. 1) [15, 20]. Two reviewers will screen all the titles, abstracts, and full texts for potential eligibility in an independent manner. Disagreements between reviewers will be resolved either by consensus or by consulting a third reviewer. In addition, the evaluation of agreement between reviewers will be tested by using Cohen’s kappa coefficient test [1].

A pilot search using the designed search strategies (Supplementary Table 1) was carried out on August 17, 2020, in which 1481 records were obtained (634 from PubMed, 674 from EMBASE, and 173 records from other sources). After the removal of duplicates (*n* = 177), 1304 unique studies were eligible for the title and abstract screening. From this first screening, 38 publications were selected for full-text screening and seven were finally selected for data extraction. Therefore, we expect to include around 10–15 studies in the final systematic review.

### Data extraction

#### General characteristics of the included articles

**Fig. 2:**
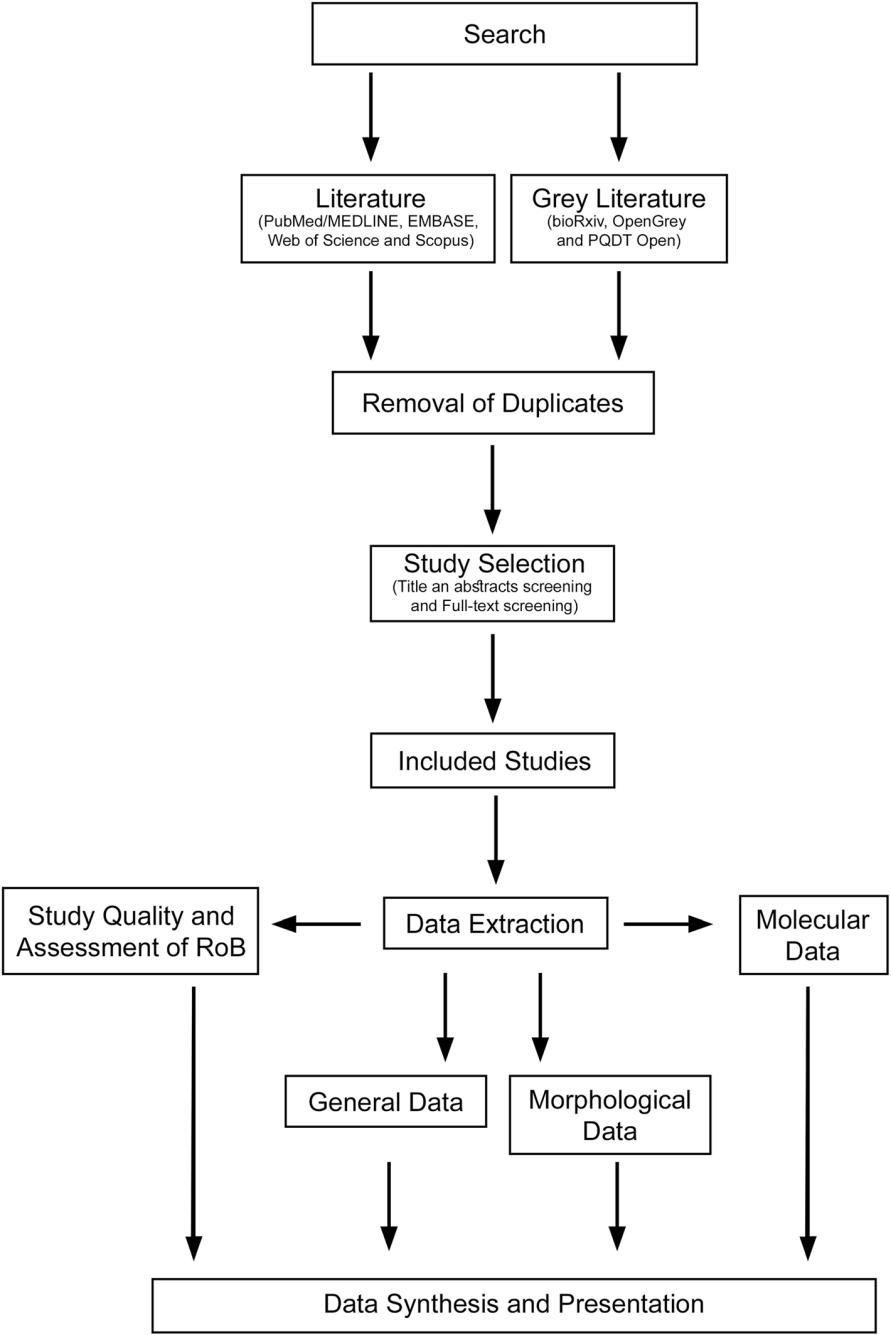
Flow chart and data processing diagram for the proposed systematic review. RoB: risk of bias

The data processing will be performed according to the diagram presented in Fig. 2. The design and methodological characteristics of each study will be extracted (Supplementary Table 2), such characteristics include type of study, sample size of each experimental group, experimental design (e.g., sample size calculation), experimental groups, animal housing, period of follow-up, age of development in which animals were inoculated, methods and techniques used for outcome assessment, and use of guidelines to conduct and publish the study (e.g., ARRIVE Statement) [9]. Information of animal models will also be extracted, including species used, lineage, age, and genetic modification factors.

#### Intervention characteristics

The method of ZIKV applications can be different among species; in some species, the teratogenic agent is applied directly onto the embryo (e.g., chicken embryos); in others, the application is applied indirectly through application to the mother (e.g., rodents and non-human primates). Therefore, the methods of viral application/embryonic exposure to ZIKV will also be extracted in detail in order to compare the different studies (Supplementary Table 2). Moreover, the amount of virus applied (volume and viral load) and the route of viral inoculation will also be extracted. Finally, the period in which animals have been exposed to ZIKV (post-inoculation time) will also be assessed individually.

#### Outcome measures

Information regarding outcome data, their units, and types will be collected according to the following outcomes:

1. Teratogenic analyses data: number and percentage of animals with congenital anomalies, descriptive summary of such anomalies, and description of organs (e.g., head and eyes) that showed differences in morphological measurements between ZIKV-exposed and controls. Since some studies have shown variability between the confirmation of infection in the mothers and the embryos/fetuses (using qPCR data), the number of animals with positive results to ZIKV infection will also be collected.
2. Molecular analyses data: the absence or presence, as well as the measurements, of altered gene expression, protein expression, epigenetic characteristics of the location of a given protein in a cell considering the experimental groups (case or controls). In addition to these descriptive data, information related to histological and cellular data will be collected. Molecular data will have their descriptive data collected (increase, decrease, or absence of variation) as well as continuous data (increase or reduction ratio, or absence of variation).

These data will be extracted from both the original articles and supplementary materials, when available or even will be requested to the authors.

Initially, data extraction will be performed by obtaining numerical and descriptive values directly from the tables, figures, and information of the results section of each study. When only percentages are presented, the values will be calculated based on the sample size information. Finally, if it is not possible to obtain relevant data, the authors will be contacted.

In cases where two research sources are potentially describing the same data, the study with a larger dataset and/or more complete reporting of the results will be included. Data collection will be conducted by one reviewer and checked by a second. Cases of disagreement will be resolved by consensus or by consultation of a third reviewer.

#### Others parameters

The number of deaths, dropouts (animals that have been removed from the study during the experiments), reason for exclusion, and incomplete experiments will be extracted.

### Assessment of risk of bias and study quality

Risk of bias and analysis of the quality of the studies will be carried out independently by two reviewers of this study. Disagreement between the review authors will be solved by including a third reviewer.

The risk of bias will be performed using the Systematic Review Center for Laboratory Animal Experimentation (SYRCLE) Risk of Bias tool developed by SYRCLE to assess animal intervention studies [8]. This tool includes domains of selection bias, driving, detection, friction, and reporting of results. The risk of bias from each study will be reported as low, unclear, and high. In addition to these types of biases evaluated, we will include in the analysis peer-reviewed publication, report on sample size calculation, and report on the use of guidelines for the execution and publication of the studies.

### Data synthesis

From the pilot searches, it was possible to observe that there is a high variability on the selected studies concerning the methods of molecular evaluation (RNAseq, qPCR, and so on), gestational period of intervention, species evaluated, etc. Therefore, a meta-analysis involving studies with such different characteristics is unlikely ever to be feasible. Therefore, data synthesis will be performed in a narrative manner, as follows.

All data obtained will be tabulated and organized by outcomes (morphological and molecular) in order to allow further analysis. The studies will also be grouped according to the used animal species for the in vivo assays, and, within the species groups, they will be differentiated in lineages and ages of embryonic/fetal development (by intervals) as well as the evaluated organs in which the molecular analyses (RT-qPCR or RNA sequencing, for example) were performed.

Morphological data will be combined and compared in a descriptive manner according to the different studies, since the focus of this review is on molecular alterations and all included studies will be those that determine structural malformations (congenital anomalies) in exposed embryos. For molecular data, a list of genes/proteins with altered expression or altered epigenetic patterns will be created according to the abovementioned grouping method. Considering that the method of evaluating these changes may have been conducted with different tools and equipment, when possible, the data will be pooled together aiming to find the most frequently affected genes/proteins. All data will be extracted from groups exposed to ZIKV and controls, and genes will be evaluated individually. Genes and proteins will be compared and ranked according to the fold change values.

The studies/data showing morphological and molecular alterations caused by ZIKV exposure during embryonic development will be prioritized for summary and synthesis. Extracted data will be first presented narratively in tables (morphological and molecular data tables) and then, whether possible, though Venn diagrams and illustrations showing common genes/proteins affected by the viral exposure.

The limitations of the data synthesis of the selected studies are related to the fact that the research question of this systematic review can generate diversified studies. For instance, the studies can utilize different animal species and different molecular analysis techniques, making their combination difficult.

### Availability of data

The complete list of studies will be made available as supplementary material, and raw data will be made available from the corresponding author on reasonable request.

## Discussion

We are conducting the first systematic review of possible molecular mechanisms involved in CZS in animal models. Nowadays, there are many studies comprising ZIKV teratogenic capacities utilizing different animal models as mice, rats, porcine, marmoset, chicken, and non-human primates [2, 3, 7, 16, 17, 21, 22, 24]. Such animal models are mainly used due to rodents being very common to biomedical research or, in case of non-human primates, due to their similarity to humans.

By compiling and comparing the data, the methodological quality of different studies on animal models will be presented. From our systematic review, other studies analyzing data in which no congenital defect was found in animals exposed to ZIKV infection during development would be interesting and helpful in explaining how slight molecular changes could explain a threshold for congenital anomalies. In this context, our results will certainly be of great relevance for future studies in this area, allowing the extrapolation of our findings in research with hypotheses directed to humans or even animal models.

## Supplementary Information

**Additional File 1: Table S1.** Draft Search Strategy. **Table S2.** List of data to be extracted from the studies included in the systematic review.

## Data Availability

The protocol of this systematic review will be available from the corresponding author on reasonable request.

## Abbreviations

SYRCLE: Center for Systematic Review for Laboratory Animal Experimentation
CZS: Congenital ZIKV Syndrome
ZIKV: Zika virus
PICO: Population, Intervention, Comparison, and Outcome
