# Supplementary material for: Molecular mechanisms of Zika virus teratogenesis from animal studies: a systematic review protocol
Source: Syst Rev. 2021 May 29;10:160. doi: 10.1186/s13643-021-01713-6 (PMC8164069; doi:10.1186/s13643-021-01713-6)
Supplement: Supplementary file 1 — Additional File 1: Table S1. Draft Search Strategy. Table S2. List of data to be extracted from the studies included in the systematic review. [file 13643_2021_1713_MOESM1_ESM.docx]

Supplementary Material

**Molecular Mechanisms of Zika Virus Teratogenesis from Animal Studies: a Systematic Review Protocol**

Gabriela Elis Wachholz, Julia do Amaral Gomes, Juliano André Boquett, Fernanda Sales Luiz Vianna, Lavínia Schuler-Faccini, Lucas Rosa Fraga

**Table S1.** Draft Search Strategy.

**Table S2.** List of data to be extracted from the studies included in the systematic review.

**Table S1.** Draft Search Strategy.

**PubMed/MEDLINE**:

#1: ZIKV:

(Zika Virus[mh] OR Zika Virus Infection[mh] OR NS1 protein, zika virus[nm] OR Zika[tiab] OR ZikV[tiab])

#2: Congenital anomalies:

(Teratogenesis[mh] OR Congenital Abnormalities[mh] OR Teratogens[mh] OR Abnormalities, Multiple[mh] OR Abnormalities, Severe Teratoid[mh] OR Heart Defects, Congenital[mh] OR Vascular Malformations[mh] OR Intracranial Arteriovenous Malformations[mh] OR Central Nervous System Vascular Malformations[mh] OR Eye Abnormalities[mh] OR Microphthalmos[mh] OR Musculoskeletal Abnormalities[mh] OR Arthrogryposis[mh] OR Craniofacial Abnormalities[mh] OR Microcephaly[mh] OR Nervous System Malformations[mh] OR Malformations of Cortical Development[mh] OR Lissencephaly[mh] OR Polymicrogyria[mh] OR Agenesis of Corpus Callosum[mh] OR Stomatognathic System Abnormalities[mh] OR Maxillofacial Abnormalities[mh] OR Dentofacial Deformities[mh] OR Micrognathism[mh] OR Retrognathia[mh] OR Tooth Abnormalities[mh] OR Teratology[mh] OR Fetal Diseases[mh] OR Fetal Growth Retardation[mh] OR Teratogen*[tiab] OR Teratolog*[tiab] OR Congenital Abnormalit*[tiab] OR Deformit*[tiab] OR Congenital Defect*[tiab] OR Birth Defect*[tiab] OR Teratoid Abnormalit*[tiab] OR Multiple Abnormalit*[tiab] OR Congenital Heart Defect*[tiab] OR Malformation Of the heart[tiab] OR Malformations Of the heart[tiab] OR Malformated heart*[tiab] OR Heart malformation*[tiab] OR Heart Abnormalit*[tiab] OR Vascular Malformation*[tiab] OR Intracranial Arteriovenous Malformation*[tiab] OR Cerebral Arteriovenous Malformation*[tiab] OR Central Nervous System Vascular Anomal*[tiab] OR Central Nervous System Congenital Vascular Malformation*[tiab] OR CNS Vascular Anomal*[tiab] OR CNS Congenital Vascular Malformation*[tiab] OR Brain Vascular Malformation*[tiab] OR Eye Abnormalit*[tiab] OR Microphthalm*[tiab] OR Musculoskeletal Abnormalit*[tiab] OR Arthrogrypos*[tiab] OR Craniofacial Abnormalit*[tiab] OR Microcephal*[tiab] OR Microlissencephal*[tiab] OR Congenital Microcephal*[tiab] OR Nervous System Malformation*[tiab] OR Nervous System Abnormalit*[tiab] OR Nervous System Anomal*[tiab] OR Nervous System Congenital Abnormalit*[tiab] OR Nervous System Congenital Malformation*[tiab] OR Malformations of Cortical Development[tiab] OR Malformations of the Cortical Development[tiab] OR Cortical Development Malformation*[tiab] OR Malformations of Cerebral Cortex Development[tiab] OR Malformations of the Cerebral Cortex Development[tiab] OR Lissencephal*[tiab] OR Agyria*[tiab] OR Pachygyria*[tiab] OR Broad Gyri of Cerebrum[tiab] OR Large Gyri of Cerebrum[tiab] OR Macrogyria*[tiab] OR Polymicrogyria*[tiab] OR Micropolygyria*[tiab] OR Cerebral Micropolygyria*[tiab] OR Cerebral Polymicrogyria*[tiab] OR Agenesis of Corpus Callosum[tiab] OR Agenesis of the Corpus Callosum[tiab] OR Corpus Callosum Agenes*[tiab] OR Corpus Callosum Dysgenes*[tiab] OR Absence of Corpus Callosum[tiab] OR Absence of the Corpus Callosum[tiab] OR Corpus Callosum Absence*[tiab] OR Corpus Callosum Hypogenes*[tiab] OR Corpus Callosum Malformation*[tiab] OR Stomatognathic System Abnormalit*[tiab] OR Maxillofacial Abnormalit*[tiab] OR Dentofacial Deformit*[tiab] OR Dentofacial Abnormalit*[tiab] OR Micrognathism[tiab] OR Mandibular Micrognathism*[tiab] OR Micrognathia*[tiab] OR Congenital Micrognathi*[tiab] OR Mandibular Micrognathia*[tiab] OR Retrognathi*[tiab] OR Tooth Abnormalit*[tiab] OR Teeth Abnormalit*[tiab] OR Fetal Disease*[tiab] OR Foetal Disease*[tiab] OR Embryopath*[tiab] OR Intrauterine Growth Retardation*[tiab] OR Intrauterine Growth Restriction*[tiab] OR Fetal Growth Restriction*[tiab] OR Foetal Growth Restriction*[tiab] OR Brain Malformation*[tiab] OR Brain Abnormalit*[tiab] OR Brain Anomal*[tiab] OR Congenital ZIKA Syndrome[tiab] OR Microencephaly[tiab])

#3: Animal Models (Animal search filter as published by SYRCLE - Hooijmans et al., 2010):

(animal experimentation[MeSH Terms] OR models, animal[MeSH Terms] OR invertebrates[MeSH Terms] OR Animals[Mesh:noexp] OR animal population groups[MeSH Terms] OR chordata[MeSH Terms:noexp] OR chordata, nonvertebrate[MeSH Terms] OR vertebrates[MeSH Terms:noexp] OR amphibians[MeSH Terms] OR birds[MeSH Terms] OR fishes[MeSH Terms] OR reptiles[MeSH Terms] OR mammals[MeSH Terms:noexp] OR primates[MeSH Terms:noexp] OR artiodactyla[MeSH Terms] OR carnivora[MeSH Terms] OR cetacea[MeSH Terms] OR chiroptera[MeSH Terms] OR elephants[MeSH Terms] OR hyraxes[MeSH Terms] OR insectivora[MeSH Terms] OR lagomorpha[MeSH Terms] OR marsupialia[MeSH Terms] OR monotremata[MeSH Terms] OR perissodactyla[MeSH Terms] OR rodentia[MeSH Terms] OR scandentia[MeSH Terms] OR sirenia[MeSH Terms] OR xenarthra[MeSH Terms] OR haplorhini[MeSH Terms:noexp] OR strepsirhini[MeSH Terms] OR platyrrhini[MeSH Terms] OR tarsii[MeSH Terms] OR catarrhini[MeSH Terms:noexp] OR cercopithecidae[MeSH Terms] OR hylobatidae[MeSH Terms] OR hominidae[MeSH Terms:noexp] OR gorilla gorilla[MeSH Terms] OR pan paniscus[MeSH Terms] OR pan troglodytes[MeSH Terms] OR pongo pygmaeus[MeSH Terms]) OR ((animals[tiab] OR animal[tiab] OR mice[Tiab] OR mus[Tiab] OR mouse[Tiab] OR murine[Tiab] OR woodmouse[tiab] OR rats[Tiab] OR rat[Tiab] OR murinae[Tiab] OR muridae[Tiab] OR cottonrat[tiab] OR cottonrats[tiab] OR hamster[tiab] OR hamsters[tiab] OR cricetinae[tiab] OR rodentia[Tiab] OR rodent[Tiab] OR rodents[Tiab] OR pigs[Tiab] OR pig[Tiab] OR swine[tiab] OR swines[tiab] OR piglets[tiab] OR piglet[tiab] OR boar[tiab] OR boars[tiab] OR sus scrofa[tiab] OR ferrets[tiab] OR ferret[tiab] OR polecat[tiab] OR polecats[tiab] OR mustela putorius[tiab] OR guinea pigs[Tiab] OR guinea pig[Tiab] OR cavia[Tiab] OR callithrix[Tiab] OR marmoset[Tiab] OR marmosets[Tiab] OR cebuella[Tiab] OR hapale[Tiab] OR octodon[Tiab] OR chinchilla[Tiab] OR chinchillas[Tiab] OR gerbillinae[Tiab] OR gerbil[Tiab] OR gerbils[Tiab] OR jird[Tiab] OR jirds[Tiab] OR merione[Tiab] OR meriones[Tiab] OR rabbits[Tiab] OR rabbit[Tiab] OR hares[Tiab] OR hare[Tiab] OR diptera[Tiab] OR flies[Tiab] OR fly[Tiab] OR dipteral[Tiab] OR drosophila[Tiab] OR drosophilidae[Tiab] OR cats[Tiab] OR cat[Tiab] OR carus[Tiab] OR felis[Tiab] OR nematoda[Tiab] OR nematode[Tiab] OR nematodes[Tiab] OR sipunculida[Tiab] OR dogs[Tiab] OR dog[Tiab] OR canine[Tiab] OR canines[Tiab] OR canis[Tiab] OR sheep[Tiab] OR sheeps[Tiab] OR mouflon[Tiab] OR mouflons[Tiab] OR ovis[Tiab] OR goats[Tiab] OR goat[Tiab] OR capra[Tiab] OR capras[Tiab] OR rupicapra[Tiab] OR rupicapras[Tiab] OR chamois[Tiab] OR haplorhini[Tiab] OR monkey[Tiab] OR monkeys[Tiab] OR anthropoidea[Tiab] OR anthropoids[Tiab] OR saguinus[Tiab] OR tamarin[Tiab] OR tamarins[Tiab] OR leontopithecus[Tiab] OR hominidae[Tiab] OR ape[Tiab] OR apes[Tiab] OR pan paniscus[Tiab] OR bonobo[Tiab] OR bonobos[Tiab] OR pan troglodytes[Tiab] OR gibbon[Tiab] OR gibbons[Tiab] OR siamang[Tiab] OR siamangs[Tiab] OR nomascus[Tiab] OR symphalangus[Tiab] OR chimpanzee[Tiab] OR chimpanzees[Tiab] OR prosimian[Tiab] OR prosimians[Tiab] OR bush baby[Tiab] OR bush babies[Tiab] OR galagos[Tiab] OR galago[Tiab] OR pongidae[Tiab] OR gorilla[Tiab] OR gorillas[Tiab] OR pongo pygmaeus[Tiab] OR orangutan[Tiab] OR orangutans[Tiab] OR lemur[Tiab] OR lemurs[Tiab] OR lemuridae[Tiab] OR horse[Tiab] OR horses[Tiab] OR equus[Tiab] OR cow[Tiab] OR calf[Tiab] OR bull[Tiab] OR chicken[Tiab] OR chickens[Tiab] OR gallus[Tiab] OR quail[Tiab] OR bird[Tiab] OR birds[Tiab] OR quails[Tiab] OR poultry[Tiab] OR poultries[Tiab] OR fowl[Tiab] OR fowls[Tiab] OR reptile[Tiab] OR reptilia[Tiab] OR reptiles[Tiab] OR snakes[Tiab] OR snake[Tiab] OR lizard[Tiab] OR lizards[Tiab] OR alligator[Tiab] OR alligators[Tiab] OR crocodile[Tiab] OR crocodiles[Tiab] OR turtle[Tiab] OR turtles[Tiab] OR amphibian[Tiab] OR amphibians[Tiab] OR amphibia[Tiab] OR frog[Tiab] OR frogs[Tiab] OR bombina[Tiab] OR salientia[Tiab] OR toad[Tiab] OR toads[Tiab] OR epidalea calamita[Tiab] OR salamander[Tiab] OR salamanders[Tiab] OR eel[Tiab] OR eels[Tiab] OR fish[Tiab] OR fishes[Tiab] OR pisces[Tiab] OR catfish[Tiab] OR catfishes[Tiab] OR siluriformes[Tiab] OR arius[Tiab] OR heteropneustes[Tiab] OR sheatfish[Tiab] OR perch[Tiab] OR perches[Tiab] OR percidae[Tiab] OR perca[Tiab] OR trout[Tiab] OR trouts[Tiab] OR char[Tiab] OR chars[Tiab] OR salvelinus[Tiab] OR minnow[Tiab] OR cyprinidae[Tiab] OR carps[Tiab] OR carp[Tiab] OR zebrafish[Tiab] OR zebrafishes[Tiab] OR goldfish[Tiab] OR goldfishes[Tiab] OR guppy[Tiab] OR guppies[Tiab] OR chub[Tiab] OR chubs[Tiab] OR tinca[Tiab] OR barbels[Tiab] OR barbus[Tiab] OR pimephales[Tiab] OR promelas[Tiab] OR poecilia reticulata[Tiab] OR mullet[Tiab] OR mullets[Tiab] OR eel[Tiab] OR eels[Tiab] OR seahorse[Tiab] OR seahorses[Tiab] OR mugil curema[Tiab] OR atlantic cod[Tiab] OR shark[Tiab] OR sharks[Tiab] OR catshark[Tiab] OR anguilla[Tiab] OR salmonid[Tiab] OR salmonids[Tiab] OR whitefish[Tiab] OR whitefishes[Tiab] OR salmon[Tiab] OR salmons[Tiab] OR sole[Tiab] OR solea[Tiab] OR lamprey[Tiab] OR lampreys[Tiab] OR pumpkinseed[Tiab] OR sunfish[Tiab] OR sunfishes[Tiab] OR tilapia[Tiab] OR tilapias[Tiab] OR turbot[Tiab] OR turbots[Tiab] OR flatfish[Tiab] OR flatfishes[Tiab] OR sciuridae[Tiab] OR squirrel[Tiab] OR squirrels[Tiab] OR chipmunk[Tiab] OR chipmunks[Tiab] OR suslik[Tiab] OR susliks[Tiab] OR vole[Tiab] OR voles[Tiab] OR lemming[Tiab] OR lemmings[Tiab] OR muskrat[Tiab] OR muskrats[Tiab] OR lemmus[Tiab] OR otter[Tiab] OR otters[Tiab] OR marten[Tiab] OR martens[Tiab] OR martes[Tiab] OR weasel[Tiab] OR badger[Tiab] OR badgers[Tiab] OR ermine[Tiab] OR mink[Tiab] OR minks[Tiab] OR sable[Tiab] OR sables[Tiab] OR gulo[Tiab] OR gulos[Tiab] OR wolverine[Tiab] OR wolverines[Tiab] OR mustela[Tiab] OR llama[Tiab] OR llamas[Tiab] OR alpaca[Tiab] OR alpacas[Tiab] OR camelid[Tiab] OR camelids[Tiab] OR guanaco[Tiab] OR guanacos[Tiab] OR chiroptera[Tiab] OR chiropteras[Tiab] OR bat[Tiab] OR bats[Tiab] OR fox[Tiab] OR foxes[Tiab] OR iguana[Tiab] OR iguanas[Tiab] OR xenopus laevis[Tiab] OR parakeet[Tiab] OR parakeets[Tiab] OR parrot[Tiab] OR parrots[Tiab] OR donkey[Tiab] OR donkeys[Tiab] OR mule[Tiab] OR mules[Tiab] OR zebra[Tiab] OR zebras[Tiab] OR shrew[Tiab] OR shrews[Tiab] OR bison[Tiab] OR bisons[Tiab] OR buffalo[Tiab] OR buffaloes[Tiab] OR deer[Tiab] OR deers[Tiab] OR bear[Tiab] OR bears[Tiab] OR panda[Tiab] OR pandas[Tiab] OR wild hog[Tiab] OR wild boar[Tiab] OR fitchew[Tiab] OR fitch[Tiab] OR beaver[Tiab] OR beavers[Tiab] OR jerboa[Tiab] OR jerboas[Tiab] OR capybara[Tiab] OR capybaras[Tiab] OR canine[tiab] OR bovine[tiab] OR porcine[tiab] OR hog[tiab] OR hogs[tiab])

#4: Combining: #1 AND #2 AND #3.

-------------------------------------------------------------------------------------------------------------------------------

**EMBASE**:

#1: ZIKV:

Zika Virus/exp OR "Zika fever"/exp OR (Zika OR ZikV):ti,ab,kw

#2: Congenital anomalies:

teratogenesis/de OR "teratogenic agent"/de OR "multiple malformation syndrome"/de OR "severe teratoid abnormality"/de OR "congenital heart malformation"/exp OR "congenital blood vessel malformation"/exp OR "brain arteriovenous malformation"/exp OR "central nervous system malformation"/exp OR "eye malformation"/exp OR "microphthalmia"/de OR "musculoskeletal system malformation"/de OR "arthrogryposis"/de OR "craniofacial malformation"/de OR "microcephaly"/de OR "nervous system malformation"/exp OR "cortical dysplasia"/exp OR "agyria"/de OR "microgyria"/de OR "corpus callosum agenesis"/de OR "mouth malformation"/exp OR "face malformation"/exp OR "dentofacial deformity"/de OR "micrognathia"/de OR "retrognathia"/de OR "tooth malformation"/exp OR "teratology"/de OR "fetus disease"/exp OR "intrauterine growth retardation"/de OR ("Teratogen*" OR "Teratolog*" OR "Congenital Abnormalit*" OR "Deformit*" OR "Congenital Defect*" OR "Birth Defect*" OR "Teratoid Abnormalit*" OR "Multiple Abnormalit*" OR "Congenital Heart Defect*" OR "Malformation Of the heart" OR "Malformations Of the heart" OR "Malformated heart*" OR "Heart malformation*" OR "Heart Abnormalit*" OR "Vascular Malformation*" OR "Intracranial Arteriovenous Malformation*" OR "Cerebral Arteriovenous Malformation*" OR "Central Nervous System Vascular Anomal*" OR "Central Nervous System Congenital Vascular Malformation*" OR "CNS Vascular Anomal*" OR "CNS Congenital Vascular Malformation*" OR "Brain Vascular Malformation*" OR "Eye Abnormalit*" OR "Microphthalm*" OR "Musculoskeletal Abnormalit*" OR "Arthrogrypos*" OR "Craniofacial Abnormalit*" OR "Microcephal*" OR "Microlissencephal*" OR "Congenital Microcephal*" OR "Nervous System Malformation*" OR "Nervous System Abnormalit*" OR "Nervous System Anomal*" OR "Nervous System Congenital Abnormalit*" OR "Nervous System Congenital Malformation*" OR "Malformations of Cortical Development" OR "Malformations of the Cortical Development" OR "Cortical Development Malformation*" OR "Malformations of Cerebral Cortex Development" OR "Malformations of the Cerebral Cortex Development" OR "Lissencephal*" OR "Agyria*" OR "Pachygyria*" OR "Broad Gyri of Cerebrum" OR "Large Gyri of Cerebrum" OR "Macrogyria*" OR "Polymicrogyria*" OR "Micropolygyria*" OR "Cerebral Micropolygyria*" OR "Cerebral Polymicrogyria*" OR "Agenesis of Corpus Callosum" OR "Agenesis of the Corpus Callosum" OR "Corpus Callosum Agenes*" OR "Corpus Callosum Dysgenes*" OR "Absence of Corpus Callosum" OR "Absence of the Corpus Callosum" OR "Corpus Callosum Absence*" OR "Corpus Callosum Hypogenes*" OR "Corpus Callosum Malformation*" OR "Stomatognathic System Abnormalit*" OR "Maxillofacial Abnormalit*" OR "Dentofacial Deformit*" OR "Dentofacial Abnormalit*" OR "Micrognathism" OR "Mandibular Micrognathism*" OR "Micrognathia*" OR "Congenital Micrognathi*" OR "Mandibular Micrognathia*" OR "Retrognathi*" OR "Tooth Abnormalit*" OR "Teeth Abnormalit*" OR "Fetal Disease*" OR "Foetal Disease*" OR "Embryopath*" OR "Intrauterine Growth Retardation*" OR "Intrauterine Growth Restriction*" OR "Fetal Growth Restriction*" OR "Foetal Growth Restriction*" OR "Brain Malformation*" OR "Brain Abnormalit*" OR "Brain Anomal*" OR "Congenital ZIKA Syndrome" OR "Microencephaly"):ti,ab,kw

#3: Animal Models (Animal search filter as published by SYRCLE - de Vries et al., 2014):

animal experiment/exp OR "animal model"/exp OR "experimental animal"/exp OR "transgenic animal"/exp OR "male animal"/exp OR "female animal"/exp OR "juvenile animal"/exp OR animal/exp OR chordata/de OR vertebrate/de OR tetrapod/de OR fish/exp OR amniote/de OR amphibia/exp OR mammal/de OR reptile/exp OR sauropsid/exp OR therian/de OR monotremate/exp OR "placental mammals"/de OR marsupial/exp OR Euarchontoglires/de OR Afrotheria/exp OR Boreoeutheria/exp OR Laurasiatheria/exp OR Xenarthra/exp OR primate/de OR Dermoptera/exp OR Glires/exp OR Scandentia/exp OR Haplorhini/de OR prosimian/exp OR simian/de OR tarsiiform/exp OR Catarrhini/de OR Platyrrhini/exp OR ape/de OR Cercopithecidae/exp OR hominid/de OR hylobatidae/exp OR chimpanzee/exp OR gorilla/exp OR orangutan/exp OR (animal OR animals OR pisces OR fish OR fishes OR catfish OR catfishes OR sheatfish OR silurus OR arius OR heteropneustes OR clarias OR gariepinus OR "fathead minnow" OR "fathead minnows" OR pimephales OR promelas OR cichlidae OR trout OR trouts OR char OR chars OR salvelinus OR salmo OR oncorhynchus OR guppy OR guppies OR millionfish OR poecilia OR goldfish OR goldfishes OR carassius OR auratus OR mullet OR mullets OR mugil OR curema OR shark OR sharks OR cod OR cods OR gadus OR morhua OR carp OR carps OR cyprinus OR carpio OR killifish OR eel OR eels OR anguilla OR zander OR sander OR lucioperca OR stizostedion OR turbot OR turbots OR psetta OR flatfish OR flatfishes OR plaice OR pleuronectes OR platessa OR tilapia OR tilapias OR oreochromis OR sarotherodon OR "common sole" OR "dover sole" OR solea OR zebrafish OR zebrafishes OR danio OR rerio OR seabass OR dicentrarchus OR labrax OR morone OR lamprey OR lampreys OR petromyzon OR pumpkinseed OR pumpkinseeds OR lepomis OR gibbosus OR herring OR clupea OR harengus OR amphibia OR amphibian OR amphibians OR anura OR salientia OR frog OR frogs OR rana OR toad OR toads OR bufo OR xenopus OR laevis OR bombina OR epidalea OR calamita OR salamander OR salamanders OR newt OR newts OR triturus OR reptilia OR reptile OR reptiles OR "bearded dragon" OR pogona OR vitticeps OR iguana OR iguanas OR lizard OR lizards OR "anguis fragilis" OR turtle OR turtles OR snakes OR snake OR aves OR bird OR birds OR quail OR quails OR coturnix OR bobwhite OR colinus OR virginianus OR poultry OR poultries OR fowl OR fowls OR chicken OR chickens OR gallus OR "zebra finch" OR taeniopygia OR guttata OR canary OR canaries OR serinus OR canaria OR parakeet OR parakeets OR grasskeet OR parrot OR parrots OR psittacine OR psittacines OR shelduck OR tadorna OR goose OR geese OR branta OR leucopsis OR woodlark OR lullula OR flycatcher OR ficedula OR hypoleuca OR dove OR doves OR geopelia OR cuneata OR duck OR ducks OR greylag OR graylag OR anser OR harrier OR "circus pygargus" OR "red knot" OR "great knot" OR calidris OR canutus OR godwit OR limosa OR lapponica OR meleagris OR gallopavo OR jackdaw OR corvus OR monedula OR ruff OR philomachus OR pugnax OR lapwing OR peewit OR plover OR vanellus OR swan OR cygnus OR columbianus OR bewickii OR gull OR chroicocephalus OR ridibundus OR albifrons OR "great tit" OR parus OR aythya OR fuligula OR streptopelia OR risoria OR spoonbill OR platalea OR leucorodia OR blackbird OR turdus OR merula OR "blue tit" OR cyanistes OR pigeon OR pigeons OR columba OR pintail OR anas OR starling OR sturnus OR owl OR "athene noctua" OR pochard OR ferina OR cockatiel OR nymphicus OR hollandicus OR skylark OR alauda OR tern OR sterna OR teal OR crecca OR oystercatcher OR haematopus OR ostralegus OR shrew OR shrews OR sorex OR araneus OR crocidura OR russula OR "european mole" OR talpa OR chiroptera OR bat OR bats OR eptesicus OR serotinus OR myotis OR dasycneme OR daubentonii OR pipistrelle OR pipistrellus OR cat OR cats OR felis OR catus OR feline OR dog OR dogs OR canis OR canine OR canines OR otter OR otters OR lutra OR badger OR badgers OR meles OR fitchew OR fitch OR foumart OR foulmart OR ferrets OR ferret OR polecat OR polecats OR mustela OR putorius OR weasel OR weasels OR fox OR foxes OR vulpes OR "common seal" OR phoca OR vitulina OR "grey seal" OR halichoerus OR horse OR horses OR equus OR equine OR equidae OR donkey OR donkeys OR mule OR mules OR pig OR pigs OR swine OR swines OR hog OR hogs OR boar OR boars OR porcine OR piglet OR piglets OR sus OR scrofa OR llama OR llamas OR lama OR glama OR deer OR deers OR cervus OR elaphus OR cow OR cows OR "bos taurus" OR "bos indicus" OR bovine OR bull OR bulls OR cattle OR bison OR bisons OR sheep OR sheeps OR "ovis aries" OR ovine OR lamb OR lambs OR mouflon OR mouflons OR goat OR goats OR capra OR caprine OR chamois OR rupicapra OR leporidae OR lagomorpha OR lagomorph OR rabbit OR rabbits OR oryctolagus OR cuniculus OR laprine OR hares OR lepus OR rodentia OR rodent OR rodents OR murinae OR mouse OR mice OR mus OR musculus OR murine OR woodmouse OR apodemus OR rat OR rats OR rattus OR norvegicus OR "guinea pig" OR "guinea pigs" OR cavia OR porcellus OR hamster OR hamsters OR mesocricetus OR cricetulus OR cricetus OR gerbil OR gerbils OR jird OR jirds OR meriones OR unguiculatus OR jerboa OR jerboas OR jaculus OR chinchilla OR chinchillas OR beaver OR beavers OR "castor fiber" OR "castor canadensis" OR sciuridae OR squirrel OR squirrels OR sciurus OR chipmunk OR chipmunks OR marmot OR marmots OR marmota OR suslik OR susliks OR spermophilus OR cynomys OR cottonrat OR cottonrats OR sigmodon OR vole OR voles OR microtus OR myodes OR glareolus OR primate OR primates OR prosimian OR prosimians OR lemur OR lemurs OR lemuridae OR loris OR "bush baby" OR "bush babies" OR bushbaby OR bushbabies OR galago OR galagos OR anthropoidea OR anthropoids OR simian OR simians OR monkey OR monkeys OR marmoset OR marmosets OR callithrix OR cebuella OR tamarin OR tamarins OR saguinus OR leontopithecus OR "squirrel monkey" OR "squirrel monkeys" OR saimiri OR "night monkey" OR "night monkeys" OR "owl monkey" OR "owl monkeys" OR douroucoulis OR aotus OR "spider monkey" OR "spider monkeys" OR ateles OR baboon OR baboons OR papio OR "rhesus monkey" OR macaque OR macaca OR mulatta OR cynomolgus OR fascicularis OR "green monkey" OR "green monkeys" OR chlorocebus OR vervet OR vervets OR pygerythrus OR hominoidea OR ape OR apes OR hylobatidae OR gibbon OR gibbons OR siamang OR siamangs OR nomascus OR symphalangus OR hominidae OR orangutan OR orangutans OR pongo OR chimpanzee OR chimpanzees OR "pan troglodytes" OR bonobo OR bonobos OR "pan paniscus" OR gorilla OR gorillas OR troglodytes):ti,ab,kw

#4: Combining:

#1 AND #2 AND #3 AND [embase]/lim NOT ([embase]/lim AND [medline]/lim) AND ('article'/it OR 'article in press'/it OR 'conference abstract'/it OR 'conference paper'/it OR 'erratum'/it OR 'letter'/it OR 'short survey'/it).

-------------------------------------------------------------------------------------------------------------------------------

**Web of Science**:

#1: ZIKV:

TS=((Zika OR ZikV)

#2: Congenital anomalies:

("Teratogen*" OR "Teratolog*" OR "Congenital Abnormalit*" OR "Deformit*" OR "Congenital Defect*" OR "Birth Defect*" OR "Teratoid Abnormalit*" OR "Multiple Abnormalit*" OR "Congenital Heart Defect*" OR "Malformation Of the heart" OR "Malformations Of the heart" OR "Malformated heart*" OR "Heart malformation*" OR "Heart Abnormalit*" OR "Vascular Malformation*" OR "Intracranial Arteriovenous Malformation*" OR "Cerebral Arteriovenous Malformation*" OR "Central Nervous System Vascular Anomal*" OR "Central Nervous System Congenital Vascular Malformation*" OR "CNS Vascular Anomal*" OR "CNS Congenital Vascular Malformation*" OR "Brain Vascular Malformation*" OR "Eye Abnormalit*" OR "Microphthalm*" OR "Musculoskeletal Abnormalit*" OR "Arthrogrypos*" OR "Craniofacial Abnormalit*" OR "Microcephal*" OR "Microlissencephal*" OR "Congenital Microcephal*" OR "Nervous System Malformation*" OR "Nervous System Abnormalit*" OR "Nervous System Anomal*" OR "Nervous System Congenital Abnormalit*" OR "Nervous System Congenital Malformation*" OR "Malformations of Cortical Development" OR "Malformations of the Cortical Development" OR "Cortical Development Malformation*" OR "Malformations of Cerebral Cortex Development" OR "Malformations of the Cerebral Cortex Development" OR "Lissencephal*" OR "Agyria*" OR "Pachygyria*" OR "Broad Gyri of Cerebrum" OR "Large Gyri of Cerebrum" OR "Macrogyria*" OR "Polymicrogyria*" OR "Micropolygyria*" OR "Cerebral Micropolygyria*" OR "Cerebral Polymicrogyria*" OR "Agenesis of Corpus Callosum" OR "Agenesis of the Corpus Callosum" OR "Corpus Callosum Agenes*" OR "Corpus Callosum Dysgenes*" OR "Absence of Corpus Callosum" OR "Absence of the Corpus Callosum" OR "Corpus Callosum Absence*" OR "Corpus Callosum Hypogenes*" OR "Corpus Callosum Malformation*" OR "Stomatognathic System Abnormalit*" OR "Maxillofacial Abnormalit*" OR "Dentofacial Deformit*" OR "Dentofacial Abnormalit*" OR "Micrognathism" OR "Mandibular Micrognathism*" OR "Micrognathia*" OR "Congenital Micrognathi*" OR "Mandibular Micrognathia*" OR "Retrognathi*" OR "Tooth Abnormalit*" OR "Teeth Abnormalit*" OR "Fetal Disease*" OR "Foetal Disease*" OR "Embryopath*" OR "Intrauterine Growth Retardation*" OR "Intrauterine Growth Restriction*" OR "Fetal Growth Restriction*" OR "Foetal Growth Restriction*" OR "Brain Malformation*" OR "Brain Abnormalit*" OR "Brain Anomal*" OR "Congenital ZIKA Syndrome")

#3: Animal Models:

(animal OR animals OR pisces OR fish OR fishes OR catfish OR catfishes OR sheatfish OR silurus OR arius OR heteropneustes OR clarias OR gariepinus OR "fathead minnow" OR "fathead minnows" OR pimephales OR promelas OR cichlidae OR trout OR trouts OR char OR chars OR salvelinus OR salmo OR oncorhynchus OR guppy OR guppies OR millionfish OR poecilia OR goldfish OR goldfishes OR carassius OR auratus OR mullet OR mullets OR mugil OR curema OR shark OR sharks OR cod OR cods OR gadus OR morhua OR carp OR carps OR cyprinus OR carpio OR killifish OR eel OR eels OR anguilla OR zander OR sander OR lucioperca OR stizostedion OR turbot OR turbots OR psetta OR flatfish OR flatfishes OR plaice OR pleuronectes OR platessa OR tilapia OR tilapias OR oreochromis OR sarotherodon OR "common sole" OR "dover sole" OR solea OR zebrafish OR zebrafishes OR danio OR rerio OR seabass OR dicentrarchus OR labrax OR morone OR lamprey OR lampreys OR petromyzon OR pumpkinseed OR pumpkinseeds OR lepomis OR gibbosus OR herring OR clupea OR harengus OR amphibia OR amphibian OR amphibians OR anura OR salientia OR frog OR frogs OR rana OR toad OR toads OR bufo OR xenopus OR laevis OR bombina OR epidalea OR calamita OR salamander OR salamanders OR newt OR newts OR triturus OR reptilia OR reptile OR reptiles OR "bearded dragon" OR pogona OR vitticeps OR iguana OR iguanas OR lizard OR lizards OR "anguis fragilis" OR turtle OR turtles OR snakes OR snake OR aves OR bird OR birds OR quail OR quails OR coturnix OR bobwhite OR colinus OR virginianus OR poultry OR poultries OR fowl OR fowls OR chicken OR chickens OR gallus OR "zebra finch" OR taeniopygia OR guttata OR canary OR canaries OR serinus OR canaria OR parakeet OR parakeets OR grasskeet OR parrot OR parrots OR psittacine OR psittacines OR shelduck OR tadorna OR goose OR geese OR branta OR leucopsis OR woodlark OR lullula OR flycatcher OR ficedula OR hypoleuca OR dove OR doves OR geopelia OR cuneata OR duck OR ducks OR greylag OR graylag OR anser OR harrier OR "circus pygargus" OR "red knot" OR "great knot" OR calidris OR canutus OR godwit OR limosa OR lapponica OR meleagris OR gallopavo OR jackdaw OR corvus OR monedula OR ruff OR philomachus OR pugnax OR lapwing OR peewit OR plover OR vanellus OR swan OR cygnus OR columbianus OR bewickii OR gull OR chroicocephalus OR ridibundus OR albifrons OR "great tit" OR parus OR aythya OR fuligula OR streptopelia OR risoria OR spoonbill OR platalea OR leucorodia OR blackbird OR turdus OR merula OR "blue tit" OR cyanistes OR pigeon OR pigeons OR columba OR pintail OR anas OR starling OR sturnus OR owl OR "athenenoctua" OR pochard OR ferina OR cockatiel OR nymphicus OR hollandicus OR skylark OR alauda OR tern OR sterna OR teal OR crecca OR oystercatcher OR haematopus OR ostralegus OR shrew OR shrews OR sorex OR araneus OR crocidura OR russula OR "european mole" OR talpa OR chiroptera OR bat OR bats OR eptesicus OR serotinus OR myotis OR dasycneme OR daubentonii OR pipistrelle OR pipistrellus OR cat OR cats OR felis OR catus OR feline OR dog OR dogs OR canis OR canine OR canines OR otter OR otters OR lutra OR badger OR badgers OR meles OR fitchew OR fitch OR foumart OR foulmart OR ferrets OR ferret OR polecat OR polecats OR mustela OR putorius OR weasel OR weasels OR fox OR foxes OR vulpes OR "common seal" OR phoca OR vitulina OR "grey seal" OR halichoerus OR horse OR horses OR equus OR equine OR equidae OR donkey OR donkeys OR mule OR mules OR pig OR pigs OR swine OR swines OR hog OR hogs OR boar OR boars OR porcine OR piglet OR piglets OR sus OR scrofa OR llama OR llamas OR lama OR glama OR deer OR deers OR cervus OR elaphus OR cow OR cows OR "bostaurus" OR "bos indicus" OR bovine OR bull OR bulls OR cattle OR bison OR bisons OR sheep OR sheeps OR "ovisaries" OR ovine OR lamb OR lambs OR mouflon OR mouflons OR goat OR goats OR capra OR caprine OR chamois OR rupicapra OR leporidae OR lagomorpha OR lagomorph OR rabbit OR rabbits OR oryctolagus OR cuniculus OR laprine OR hares OR lepus OR rodentia OR rodent OR rodents OR murinae OR mouse OR mice OR mus OR musculus OR murine OR woodmouse OR apodemus OR rat OR rats OR rattus OR norvegicus OR "guinea pig" OR "guinea pigs" OR cavia OR porcellus OR hamster OR hamsters OR mesocricetus OR cricetulus OR cricetus OR gerbil OR gerbils OR jird OR jirds OR meriones OR unguiculatus OR jerboa OR jerboas OR jaculus OR chinchilla OR chinchillas OR beaver OR beavers OR "castor fiber" OR "castor canadensis" OR sciuridae OR squirrel OR squirrels OR sciurus OR chipmunk OR chipmunks OR marmot OR marmots OR marmota OR suslik OR susliks OR spermophilus OR cynomys OR cottonrat OR cottonrats OR sigmodon OR vole OR voles OR microtus OR myodes OR glareolus OR primate OR primates OR prosimian OR prosimians OR lemur OR lemurs OR lemuridae OR loris OR "bush baby" OR "bush babies" OR bushbaby OR bushbabies OR galago OR galagos OR anthropoidea OR anthropoids OR simian OR simians OR monkey OR monkeys OR marmoset OR marmosets OR callithrix OR cebuella OR tamarin OR tamarins OR saguinus OR leontopithecus OR "squirrel monkey" OR "squirrel monkeys" OR saimiri OR "night monkey" OR "night monkeys" OR "owl monkey" OR "owl monkeys" OR douroucoulis OR aotus OR "spider monkey" OR "spider monkeys" OR ateles OR baboon OR baboons OR papio OR "rhesus monkey" OR macaque OR macaca OR mulatta OR cynomolgus OR fascicularis OR "green monkey" OR "green monkeys" OR chlorocebus OR vervet OR vervets OR pygerythrus OR hominoidea OR ape OR apes OR hylobatidae OR gibbon OR gibbons OR siamang OR siamangs OR nomascus OR symphalangus OR hominidae OR orangutan OR orangutans OR pongo OR chimpanzee OR chimpanzees OR "pan troglodytes" OR bonobo OR bonobos OR "pan paniscus" OR gorilla OR gorillas OR troglodytes)

#4: Combining:

#1 AND #2 AND #3

-------------------------------------------------------------------------------------------------------------------------------

**Scopus**:

#1: ZIKV:

TITLE-ABS-KEY(Zika OR ZikV)

#2: Congenital anomalies:

TITLE-ABS-KEY("Teratogen*" OR "Teratolog*" OR "Congenital Abnormalit*" OR "Deformit*" OR "Congenital Defect*" OR "Birth Defect*" OR "Teratoid Abnormalit*" OR "Multiple Abnormalit*" OR "Congenital Heart Defect*" OR "Malformation Of the heart" OR "Malformations Of the heart" OR "Malformated heart*" OR "Heart malformation*" OR "Heart Abnormalit*" OR "Vascular Malformation*" OR "Intracranial Arteriovenous Malformation*" OR "Cerebral Arteriovenous Malformation*" OR "Central Nervous System Vascular Anomal*" OR "Central Nervous System Congenital Vascular Malformation*" OR "CNS Vascular Anomal*" OR "CNS Congenital Vascular Malformation*" OR "Brain Vascular Malformation*" OR "Eye Abnormalit*" OR "Microphthalm*" OR "Musculoskeletal Abnormalit*" OR "Arthrogrypos*" OR "Craniofacial Abnormalit*" OR "Microcephal*" OR "Microlissencephal*" OR "Congenital Microcephal*" OR "Nervous System Malformation*" OR "Nervous System Abnormalit*" OR "Nervous System Anomal*" OR "Nervous System Congenital Abnormalit*" OR "Nervous System Congenital Malformation*" OR "Malformations of Cortical Development" OR "Malformations of the Cortical Development" OR "Cortical Development Malformation*" OR "Malformations of Cerebral Cortex Development" OR "Malformations of the Cerebral Cortex Development" OR "Lissencephal*" OR "Agyria*" OR "Pachygyria*" OR "Broad Gyri of Cerebrum" OR "Large Gyri of Cerebrum" OR "Macrogyria*" OR "Polymicrogyria*" OR "Micropolygyria*" OR "Cerebral Micropolygyria*" OR "Cerebral Polymicrogyria*" OR "Agenesis of Corpus Callosum" OR "Agenesis of the Corpus Callosum" OR "Corpus Callosum Agenes*" OR "Corpus Callosum Dysgenes*" OR "Absence of Corpus Callosum" OR "Absence of the Corpus Callosum" OR "Corpus Callosum Absence*" OR "Corpus Callosum Hypogenes*" OR "Corpus Callosum Malformation*" OR "Stomatognathic System Abnormalit*" OR "Maxillofacial Abnormalit*" OR "Dentofacial Deformit*" OR "Dentofacial Abnormalit*" OR "Micrognathism" OR "Mandibular Micrognathism*" OR "Micrognathia*" OR "Congenital Micrognathi*" OR "Mandibular Micrognathia*" OR "Retrognathi*" OR "Tooth Abnormalit*" OR "Teeth Abnormalit*" OR "Fetal Disease*" OR "Foetal Disease*" OR "Embryopath*" OR "Intrauterine Growth Retardation*" OR "Intrauterine Growth Restriction*" OR "Fetal Growth Restriction*" OR "Foetal Growth Restriction*" OR "Brain Malformation*" OR "Brain Abnormalit*" OR "Brain Anomal*" OR "Congenital ZIKA Syndrome")

#3: Animal Models:

TITLE-ABS-KEY(animal OR animals OR pisces OR fish OR fishes OR catfish OR catfishes OR sheatfish OR silurus OR arius OR heteropneustes OR clarias OR gariepinus OR "fathead minnow" OR "fathead minnows" OR pimephales OR promelas OR cichlidae OR trout OR trouts OR char OR chars OR salvelinus OR salmo OR oncorhynchus OR guppy OR guppies OR millionfish OR poecilia OR goldfish OR goldfishes OR carassius OR auratus OR mullet OR mullets OR mugil OR curema OR shark OR sharks OR cod OR cods OR gadus OR morhua OR carp OR carps OR cyprinus OR carpio OR killifish OR eel OR eels OR anguilla OR zander OR sander OR lucioperca OR stizostedion OR turbot OR turbots OR psetta OR flatfish OR flatfishes OR plaice OR pleuronectes OR platessa OR tilapia OR tilapias OR oreochromis OR sarotherodon OR "common sole" OR "dover sole" OR solea OR zebrafish OR zebrafishes OR danio OR rerio OR seabass OR dicentrarchus OR labrax OR morone OR lamprey OR lampreys OR petromyzon OR pumpkinseed OR pumpkinseeds OR lepomis OR gibbosus OR herring OR clupea OR harengus OR amphibia OR amphibian OR amphibians OR anura OR salientia OR frog OR frogs OR rana OR toad OR toads OR bufo OR xenopus OR laevis OR bombina OR epidalea OR calamita OR salamander OR salamanders OR newt OR newts OR triturus OR reptilia OR reptile OR reptiles OR "bearded dragon" OR pogona OR vitticeps OR iguana OR iguanas OR lizard OR lizards OR "anguis fragilis" OR turtle OR turtles OR snakes OR snake OR aves OR bird OR birds OR quail OR quails OR coturnix OR bobwhite OR colinus OR virginianus OR poultry OR poultries OR fowl OR fowls OR chicken OR chickens OR gallus OR "zebra finch" OR taeniopygia OR guttata OR canary OR canaries OR serinus OR canaria OR parakeet OR parakeets OR grasskeet OR parrot OR parrots OR psittacine OR psittacines OR shelduck OR tadorna OR goose OR geese OR branta OR leucopsis OR woodlark OR lullula OR flycatcher OR ficedula OR hypoleuca OR dove OR doves OR geopelia OR cuneata OR duck OR ducks OR greylag OR graylag OR anser OR harrier OR "circus pygargus" OR "red knot" OR "great knot" OR calidris OR canutus OR godwit OR limosa OR lapponica OR meleagris OR gallopavo OR jackdaw OR corvus OR monedula OR ruff OR philomachus OR pugnax OR lapwing OR peewit OR plover OR vanellus OR swan OR cygnus OR columbianus OR bewickii OR gull OR chroicocephalus OR ridibundus OR albifrons OR "great tit" OR parus OR aythya OR fuligula OR streptopelia OR risoria OR spoonbill OR platalea OR leucorodia OR blackbird OR turdus OR merula OR "blue tit" OR cyanistes OR pigeon OR pigeons OR columba OR pintail OR anas OR starling OR sturnus OR owl OR "athenenoctua" OR pochard OR ferina OR cockatiel OR nymphicus OR hollandicus OR skylark OR alauda OR tern OR sterna OR teal OR crecca OR oystercatcher OR haematopus OR ostralegus OR shrew OR shrews OR sorex OR araneus OR crocidura OR russula OR "european mole" OR talpa OR chiroptera OR bat OR bats OR eptesicus OR serotinus OR myotis OR dasycneme OR daubentonii OR pipistrelle OR pipistrellus OR cat OR cats OR felis OR catus OR feline OR dog OR dogs OR canis OR canine OR canines OR otter OR otters OR lutra OR badger OR badgers OR meles OR fitchew OR fitch OR foumart OR foulmart OR ferrets OR ferret OR polecat OR polecats OR mustela OR putorius OR weasel OR weasels OR fox OR foxes OR vulpes OR "common seal" OR phoca OR vitulina OR "grey seal" OR halichoerus OR horse OR horses OR equus OR equine OR equidae OR donkey OR donkeys OR mule OR mules OR pig OR pigs OR swine OR swines OR hog OR hogs OR boar OR boars OR porcine OR piglet OR piglets OR sus OR scrofa OR llama OR llamas OR lama OR glama OR deer OR deers OR cervus OR elaphus OR cow OR cows OR "bostaurus" OR "bos indicus" OR bovine OR bull OR bulls OR cattle OR bison OR bisons OR sheep OR sheeps OR "ovisaries" OR ovine OR lamb OR lambs OR mouflon OR mouflons OR goat OR goats OR capra OR caprine OR chamois OR rupicapra OR leporidae OR lagomorpha OR lagomorph OR rabbit OR rabbits OR oryctolagus OR cuniculus OR laprine OR hares OR lepus OR rodentia OR rodent OR rodents OR murinae OR mouse OR mice OR mus OR musculus OR murine OR woodmouse OR apodemus OR rat OR rats OR rattus OR norvegicus OR "guinea pig" OR "guinea pigs" OR cavia OR porcellus OR hamster OR hamsters OR mesocricetus OR cricetulus OR cricetus OR gerbil OR gerbils OR jird OR jirds OR meriones OR unguiculatus OR jerboa OR jerboas OR jaculus OR chinchilla OR chinchillas OR beaver OR beavers OR "castor fiber" OR "castor canadensis" OR sciuridae OR squirrel OR squirrels OR sciurus OR chipmunk OR chipmunks OR marmot OR marmots OR marmota OR suslik OR susliks OR spermophilus OR cynomys OR cottonrat OR cottonrats OR sigmodon OR vole OR voles OR microtus OR myodes OR glareolus OR primate OR primates OR prosimian OR prosimians OR lemur OR lemurs OR lemuridae OR loris OR "bush baby" OR "bush babies" OR bushbaby OR bushbabies OR galago OR galagos OR anthropoidea OR anthropoids OR simian OR simians OR monkey OR monkeys OR marmoset OR marmosets OR callithrix OR cebuella OR tamarin OR tamarins OR saguinus OR leontopithecus OR "squirrel monkey" OR "squirrel monkeys" OR saimiri OR "night monkey" OR "night monkeys" OR "owl monkey" OR "owl monkeys" OR douroucoulis OR aotus OR "spider monkey" OR "spider monkeys" OR ateles OR baboon OR baboons OR papio OR "rhesus monkey" OR macaque OR macaca OR mulatta OR cynomolgus OR fascicularis OR "green monkey" OR "green monkeys" OR chlorocebus OR vervet OR vervets OR pygerythrus OR hominoidea OR ape OR apes OR hylobatidae OR gibbon OR gibbons OR siamang OR siamangs OR nomascus OR symphalangus OR hominidae OR orangutan OR orangutans OR pongo OR chimpanzee OR chimpanzees OR "pan troglodytes" OR bonobo OR bonobos OR "pan paniscus" OR gorilla OR gorillas OR troglodytes)

#4: Combining:

#1 AND #2 AND #3

-------------------------------------------------------------------------------------------------------------------------------

**REFERENCES**:

Hooijmans CR, Tillema A, Leenaars M, Ritskes-Hoitinga M (2010) Enhancing search efficiency by means of a search filter for finding all studies on animal experimentation in PubMed. Lab Anim. 44(3): p. 170-5.

de Vries RBM, Hooijmans CR, Tillema A, Leenaars M, Ritskes-Hoitinga M (2014) Updated version of the Embase search filter for animal studies. Lab Anim. Jan;48(1):88.

| **Supplementary Table 2**. List of data to be extracted from the studies included in the systematic review. |
| --- |
| Author |
| Title |
| Year of publication |
| DOI |
| Type of study |
| Experimental groups |
| Sample size per group |
| Was there a sample size estimation? |
| Animals husbandry |
| Follow-up period |
| Developmental stage on the infection |
| Method of outcome evaluation |
| Use of guidelines? |
| Animal species |
| Animal strain |
| Genetically modified? |
| ZIKV lineage |
| Developmental stage on the outcome evaluation |
| Method of viral inoculation |
| Amount of virus inoculated |
| Morphological outcomes in details |
| Type of molecular analysis |
| Molecular alterations found |
| Genes/Proteins affected |
| Measure of increase/decreased for each gene |
| Number of deaths |
| Number of excluded animals |
| Reason for excluding |
| Number and % of malformed animals for each experimental group |
| Organs where malformations were observed |
| Developmental stage when malformations occurred |
